# Probiotics mediated gut microbiota diversity shifts are associated with reduction in histopathology and shedding of *Lawsonia intracellularis*

**DOI:** 10.1186/s42523-021-00084-6

**Published:** 2021-03-04

**Authors:** Adrian Muwonge, Anbu K. Karuppannan, Tanja Opriessnig

**Affiliations:** 1 The Roslin Institute and The Royal (Dick) School of Veterinary Studies, University of Edinburgh, Midlothian, UK; 2 Vaccine Research Centre-Viral Vaccines, Centre for Animal Health Studies, Tamil Nadu Veterinary and Animal Sciences University, Chennai, India; 3 Department of Veterinary Diagnostic and Production Animal Medicine, College of Veterinary Medicine, Iowa State University, Ames, IA USA

**Keywords:** *Lawsonia intracellularis*, Pigs, Probiotics, Vaccine, Microbial community, Metagenomics

## Abstract

**Background:**

Clinical intervention during bacterial infections in farm animals such as pigs commonly includes the use of antimicrobials. With the rise of antimicrobial resistance and the attempts to reduce the use of antibiotics in food animals, effective alternatives are urgently needed to reduce or even remove pathogens and disease risks. Improving clinical outcomes and overall pig health by using probiotics appears attractive. However, reliable data sets on the efficacy of probiotics are scarce. The obligate intracellular bacterium *Lawsonia intracellularis* is widespread in pigs and associated with severe enteropathy, mainly in the ileum, commonly resulting in substantial reduction in weight gain. The impact of three in-feed probiotics and a commercial live *L. intracellularis* vaccine was compared in a pig challenge model. Probiotic treatment was associated with reduced *L. intracellularis* fecal shedding and reduced gut lesions. Here, the bacterial microbiota of the ileum of these pigs was characterized with 16S rRNA gene sequencing and was subsequently analyzed with bioinformatics tools.

**Results:**

The greatest microbial richness was observed in the probiotic treated group T03-LAW, which accounted for 87% of richness observed in the study. Treatment had a significant impact on both the microbiota structure and taxonomic profile in the ileum, explaining between 26 and 36% of the structural variation, with the strongest association in the T03-LAW group. Overall, the largest changes were observed for the pigs treated with in-feed *Bacillus pumilus*; the microbiota of these pigs had the greatest diversity and highest richness. We also observed depleted and enriched core microbiota amongst the groups; however, there was no correlation with clinical characteristics. The results suggest that an increased diversity of the ileal microbiota is associated with a reduction in shedding, i.e. a unit increase in Shannon diversity index resulted in 2.8 log reduction in shedding.

**Conclusions:**

Probiotic supplementation of a base feed ration increased ileum microbiota diversity leading to a mitigation of the effects of a pathogenic *L. intracellularis* challenge. An even and diverse microbiota community benefits pigs infected with *L. intracellularis*, however, investigations are needed to determine if this is also true for other pathogens. The study unambiguously demonstrates the usefulness of probiotic supplementation in reducing the impact of enteric pathogens and pathogen shedding rates in food animals without the use of antimicrobials.

**Supplementary Information:**

The online version contains supplementary material available at 10.1186/s42523-021-00084-6.

## Background

Probiotics are increasingly used in commercial food animal production and are also slowly being implemented in pork production. Interactions of diet, gut microbiota, and host appear to impact animal health and disease [1]. Common feed additives, such as dietary phytase supplementation and lactic acid treatment of cereals, drastically altered the bacterial community composition in feces of growing pigs [2]. Probiotic supplementation is thought to have a beneficial effect for pigs, however, its impact on the gut microbiome itself is less clear and is yet to be robustly investigated. Limited numbers of studies have investigated the porcine gut microbiota in the context of dietary changes. Dietary phytase supplementation affected all major bacterial taxa, but reduced species richness and diversity in fattening pigs based on 16S rRNA gene sequencing of fecal DNA [3]. Shifts in the composition of the fecal microflora, in response to the administration of prebiotic lactulose, significantly increased the *Firmicutes*-to-*Bacteroidetes* ratio in weaned pigs [4]. At the family level, *Lactobacillaceae* numbers were increased after administration of the prebiotic lactulose [4]. Probiotic groups in general were found to have a lower relative abundance of *Escherichia coli*, suggesting that the gut microbiome of piglets may have shifted functionally and mechanistically [5]. Antimicrobials, regardless of global concerns for antibiotic resistance, are still widely used in pig farming as prophylaxis for bacterial diseases and as growth promoters in addition to therapeutic purposes [6]. Feasible and cost-effective alternatives such as probiotics, which hold much promise, are urgently needed.

*Lawsonia intracellularis (L. intracellularis),* an obligate intracellular bacterium with a tropism for intestinal epithelial cells, especially in the ileum [7], is of great importance in pig production [8]. In pigs, *L. intracellularis* is associated with ileitis, also known as proliferative enteritis, which manifests as an acute or chronic form [7]. In addition, subclinical infection of pigs is common and often remains unnoticed while impacting production and economic cost [9]. Transmission of *L. intracellularis* among pigs occurs by the oral fecal route [10], most pig herds are infected [8] and the disease may be exacerbated by co-infecting pathogens [11–14]. The high occurrence of *L. intracellularis* infection after weaning has been associated with microbial dysbiosis resulting from the change in feeding regiments [15]. Similarly, *L. intracellularis* infection is also known to affect the gut microbiome [11, 15, 16].

Control of *L. intracellularis* infection is often accomplished by either vaccination or antimicrobial treatment. High doses of antibiotics such as tiamulin, tylosin, chlortetracycline, lincomycin and olaquindox have been shown to be effective [17, 18]. Oral live and parenteral inactivated vaccines are available to control *L. intracellularis* [19, 20]. In vaccinated pigs, *L. intracellularis* shedding is not completely eliminated, regardless of vaccine type or vaccination route [21, 22]. A recent Danish study found no clear beneficial effect of vaccination against common swine pathogens including *L. intracellularis* and the amount of prescribed antimicrobials [23]. Moreover, there is no study to date on the effect, if any, of vaccines on the dysbiosis caused by *L. intracellularis* infection. In addition, there is limited knowledge on the effect of live attenuated *L. intracellularis* vaccine on the microbiota of the intestine [11].

In a previous controlled *L. intracellular*is challenge study we demonstrated  changes in a) time to onset of clinical pathology, b) levels of shedding and c) severity of shedding associated with a commercial live *L. intracellularis* vaccine supplemented via feed with one of three *Bacillus* strains and control (non-supplemented/non-vaccinated) compared to non-treated infected pigs [24]. Here we examine the changes in the aforementioned groups in relation to microbiota structure of the ileum using archived ileum content in a follow-up study. Microbiota structure and taxonomic characteristics were profiled using 16S rRNA gene amplicon sequencing with primers targeting the prokaryotic hypervariable region 4 (V4).

## Methods

### Experiment design

Details on the experimental design (Table 1) and clinical findings and *L. intracellularis* infection characteristics including pathology and shedding results unrelated to this investigation have been published [24]. In brief, 3-week-old cross-bred pigs (the paternal line was a Hampshire descent i.e. PIC®327, while the maternal line was based on Landrace/Large White descents i.e. FAST 276) were randomly assigned to one of six treatment groups with 10–20 pigs each. The pigs were housed in four separate BSL-2 rooms with 1–3 pens (approximately 10 m^2^ each) of 10 pigs each equipped with a nipple waterer and a self-feeder. Specifically, the T01-LAW, T02-LAW and T03-LAW pigs were housed in a single room but in three separate pens approximately 2 m apart from each other. Similarly, the POS-CONTROL group was also housed in a single room in two separate pens. The NEG-CONTROL and VAC-LAW pigs were housed in separate single rooms with a single pen each [24]. At 3 weeks of age, the pigs were either vaccinated with a commercial oral vaccine against *L. intracellularis* (VAC-LAW, *n* = 10), were supplied feed supplemented with one of the three *Bacillus* probiotics (T01-LAW, *B. amyloliquefaciens*, n = 10; T02-LAW, *B. licheniformis*, n = 10; T03-LAW, *B. pumilus*, n = 10), or remained non-treated (NEG-CONTROL, n = 10; POS-CONTROL, *n* = 20). At 7 weeks of age all groups, excluding the NEG-CONTROL pigs, were challenged with gut homogenate containing *L. intracellularis* by gastric gavage. The gut homogenate, obtained from a commercial vendor, was produced by collecting intestines from a field pig suffering from ileitis. The affected mucosa was collected and processed (i.e. grinded and diluted), and the resulting homogenate was passaged in experimental specific pathogen free pigs to obtain mucosal scrapings with a high *L. intracellularis* load free of other common pig pathogens. A homogenized stock was used for inoculation of all pigs to guarantee that each pigs was equally exposed to microbial loads that potentially could have been present in the inoculum. All pigs were euthanized 16 days post *L. intracellularis* challenge, at approximately 9 weeks old [24].

**Table 1:** Experimental design

Treatment group	Number of pigs	Feed information (administered for the entire study duration)	*Key events*		
			Vaccination 3 weeks of age − 28 dpc^b^	Challenge 7 weeks of age 0 dpc	Necropsy 9 weeks of age 16 dpc
T01-LAW	10	Base diet + *Bacillus amyloliquefaciens*^a^	None	*Lawsonia intracellularis* challenge using a gut homogenate	Ileum content collection for this study
T02-LAW	10	Base diet + *Bacillus licheniformis*^a^	None		
T03-LAW	10	Base diet + *Bacillus pumilus*^a^	None		
VAC-LAW	10	Base diet only	Commercial vaccine administration^c^		
POS-CONTROL	20	Base diet only	None		
NEG-CONTROL	10	Base diet only	None	None	

^a^ Supplemented in the feed mill with 1 × 10^12^ colony forming units (CFU) of the respective *Bacillus* strain

^b^ Day post *Lawsonia intracellularis* challenge

^c^ Enterisol® Ileitis, Boehringer Ingelheim, serial number 3040187B, via the oral route by drenching 2 ml of the vaccine, reconstituted as per manufacturer’s instructions, into the mouth of each pig

### Measuring *L. intracellularis* infection kinetics

*L. intracellularis* shedding was evaluated by measuring the bacterial load in rectal swabs. Rectal swabs were collected at dpc 0, 2, 4, 6, 8, 10, 12 and 15 using polyester swabs and stored in 5 mL plastic tubes containing 1 mL of sterile saline solution at − 80 °C until testing. Testing was done using a quantitative real-time PCR specific for *L. intracellularis* as described [24]. Prior to analysis, genomic copy numbers were log_10_ transformed to normalize them. These values were used to calculate the total area under the curve (AUC) for each group, one of the outcomes utilized in this analysis. *L. intracellularis* associated histopathology was described using a combined ileum lesion score based on microscopic lesions and amount of *L. intracellularis* antigen within lesions. In brief, individual scores of crypt enterocyte hyperplasia (range from 0 = normal to 3 = marked with or without crypt herniation into the submucosa), inflammation (range from 0 = normal to 3 = marked cellular infiltrate with or without submucosal infiltrate) and amount of *L. intracellularis* antigen as determined by immunohistochemistry (range 0 = no signal to 3 = most crypts in most sessions with marked apical enterocyte labelling) were combined for a maximal score of 9 [24].

### Sample collection and sequencing

At necropsy, luminal ileum content was collected from each pig approximately 10 cm anterior to the ileocecal junction and stored at − 80 °C until use. In addition, the inoculum stock, used to infect the pigs, was also processed and sequenced. Approximately 1 g (wet weight) of the luminal ileum content from each pig or 1 g of the challenge material was used to extract genomic DNA using PowerMag DNA Isolation Kit (MO BIO Laboratories, Inc. Carlsbad, CA, USA) following the manufacturer’s instructions. The quality of the DNA was assessed by Qubit 4 Fluorometer (ThermoFisher, Waltham, MA, USA). The 16S rRNA amplicon library was prepared as described [25] with minor modifications. Briefly, the V4 region of the 16S rRNA gene was sequenced using the Illumina MiSeq sequencing platform with v2 MiSeq cartridges to produce 2 × 250 bp paired end reads. DNA was amplified by using the 515f/806r primer set forward V4 (GTGCCAGCMGCCGCGGTAA) and reverse V4 (GGACTACHVGGGTWTCTAAT). The primers for library preparation were designed based on the V4 primers, multiplex indices and Illumina adapter sequences. Extracted genomic DNA was PCR amplified with Schloss lab indices and AccuPrime™ Pfx SuperMix (ThermoFisher, Waltham, MA, USA) using the following cycling conditions: initial denaturation at 95 °C for 2 min followed by 30 cycles of denaturation at 95 °C for 20 s, annealing at 55 °C for 15 s, extension at 72 °C for 5 min, and a final extension at 72 °C for 10 min. The prepared library was purified using Agencourt AMPure XP beads, (Beckman Coulter®, Brea, CA, USA). Libraries were quantified using the Qubit dsDNA HS assay kit (ThermoFisher, Waltham, MA, USA). The pooled library was fed into the sequencing workflow with 15% PhiX control library at a final concentration of 4pM, using MiSeq Reagent Nano kit v2 (Illumina, San Diego, CA, USA) for a 500 cycle (2 × 250 bp) run. Custom sequencing primers designed on the V4 region were utilized during the sequencing procedure, together with MiSeq sequencing primers (Illumina, San Diego, CA, USA) as follows: read 1 primer (TATGGTAATTGTGTGCCAGCMGCCGCGGTAA), read 2 primer (AGTCAGTCAGCCGGACTACHVGGGTWTCTAAT) and index primer (ATTAGAWACCCBDGTAGTCCGGCTGACTGACT). Raw data was de-multiplexed based on dual indices to generate two FASTQ files, R1 and R2, for each sample.

### Sequence processing using QIIME-2

The paired end reads from the sequencing step above were processed using the Quantitative Insights Into Microbial Ecology-2™ (QIIME-2) software [26]. The raw reads and the corresponding experimental metadata were combined using a manifest generated QIIME-2 analysis file. The reads were then de-replicated and chimeric sequences were removed before the denoising was done in DADA2 [26]. The resultant feature tables were used to identify the appropriate sequence depth at which the subsequent analyses would be performed. In addition, output data from a rarefaction analysis step was used to identify and remove spurious sequences at the appropriate sequence depth. Alpha and beta diversity indices as well as the operational taxonomic unit (OTU) phylogenetic were obtained by following the QIIME-2 workflow [26]. OTU taxonomic classification was done using a naïve Bayes classifier trained on the SILVA database (vMarch 2020) at 97% similarity [27]. First, a training dataset using the sequencing primers was extracted. The classifier was then used after the training to assign taxonomy to our OTU dataset.

### Ileal microbiota structure analysis

The analysis of the microbiota community structure was based on characteristics of the alpha and beta diversities. In brief, data from QIIME-2 was exported into the R statistical package (v3.5.1). Four files, including the taxonomic map, the OTU biome table, the experimental metadata and the rooted phylogenetic tree, were combined to generate a *phyloseq* object [28] which was used for downstream analysis. To analyze the alpha diversity, we first normalized the taxa abundance using the “relative” option in the normalize_data function from the “microbiome” R package [29]. Then the plot_anova-diversity function from the same package was used with the adjusted level of significance set at ≤0.05 for species richness (observed OTU count), the Simpson index and the Shannon index [30]. Pairwise comparison between treatment and each alpha index was done using the non-parametric Wilcoxon test with a significant *p*-value set at < 0.05 [31]. We also explored four beta diversity indices, i.e. Bray-Curtis dissimilarity, Jensen-Shannon divergence (JSD), double principal coordinate analysis (DPCoA), and weighted UniFrac distances, to characterize the impact of treatment on microbiota structure [32–34]. Each of these beta diversity indices exploits slightly different aspects of the microbiota structure and a comparative examination of these tools allowed us to determine the extent to which community structural variations are explained by treatment or sex.

### Treatment or sex associated microbial structural changes

To assess multivariate associations of experimental factors and microbial structural changes, a permutation multivariate analysis of variance (PerMANOVA) model with the Adonis function (9999 permutations) was used in the “microbiome” package in R. Here a model was run for each of the beta diversity indices with the aim to assess the individual and cumulative variation explained by the factors (sex, treatment) used in this study.

### Taxonomic profiles of ileal microbiota

The “metacoder” package (v0.3.2) in R [35] was used to profile the ileal microbial taxonomy, and taxonomic assignments (pan-phylogenetic) and their abundances (heat tree) were plotted based on treatment. This allowed for visualization of changes in abundance of each phylogenetic tree branches in each treatment group. In addition, mean abundances per family were computed and visualized using the “ComplexHeatmap” package (v2.5.1) as described [36]. The assumption for this analysis was if the hierarchical clustering of taxa recapitulates the treatment groups, then there should be a strong relationship between the taxonomic profiles and treatment groups [37].

### Comparison between *L. intracellularis* clinical characteristics and ileal microbiota

To identify any relationship between the microbiota characteristics and the clinical signs of infection, i.e. histopathology and fecal shedding of *L. intracellularis* [24], we used a correlation analysis and the multinomial logistic regression to fit a model using the “ggbupr*”* package (v0.4.0) in R. First, we assessed the correlation between shedding and histopathology, then examined how each is associated with primary microbiota parameters such as abundance, diversity and richness as well as secondary parameters such as clustering. Parameters and characteristics were defined as directly or inversely correlated and a statistical significance of *p* < 0.05 determined the significance of the association. This allowed us to identify treatment-mediated parameters associated with reduced shedding and histopathology.

## Results

### Summary description of the study group

A total of 70 pigs, sero-negative for *L. intracellularis*, were included. Each group contained 10 animals, excluding the POS-CONTROL group which contained 20 animals. The pigs were purchased from a commercial farm in the Midwest of the US and were cross-bred pigs. Overall, 54.3% (38/70) of the pigs were male and 45.7% (32/70) were female. Samples used for this study were collected from 9-week-old pigs of which the majority had been experimentally challenged with *L. intracellularis* 16 days earlier. They spent 44 days on their specific diet with or without probiotic supplementation.

### Infection kinetics and pathology associated with *L. intracellularis*

On arrival, all pigs were free of *L. intracellularis* antibodies and DNA as determined by ELISA on serum and PCR on fecal samples [24]. By the time of inoculation 4 weeks after *L. intracellularis* vaccination, all VAC-LAW pigs had seroconverted and, at necropsy, 10–65% of the pigs in all other challenged groups were also seropositive. Successful *L. intracellularis* challenge was confirmed by a high prevalence of pigs shedding *L. intracellularis* with the highest bacterial DNA levels detected in POS-CONTROL pigs, VAC-LAW pigs and T01-LAW pigs (Additional file 1). There was a delay in onset of shedding in T02-LAW and T03-LAW groups, which was reflected in less severe macroscopic and microscopic lesions (Additional file 2), reduced intralesional *L. intracellularis* antigen levels and a lower area under the curve for bacterial shedding [24].

### 16S rRNA gene sequencing output statistics

**Fig. 1:**
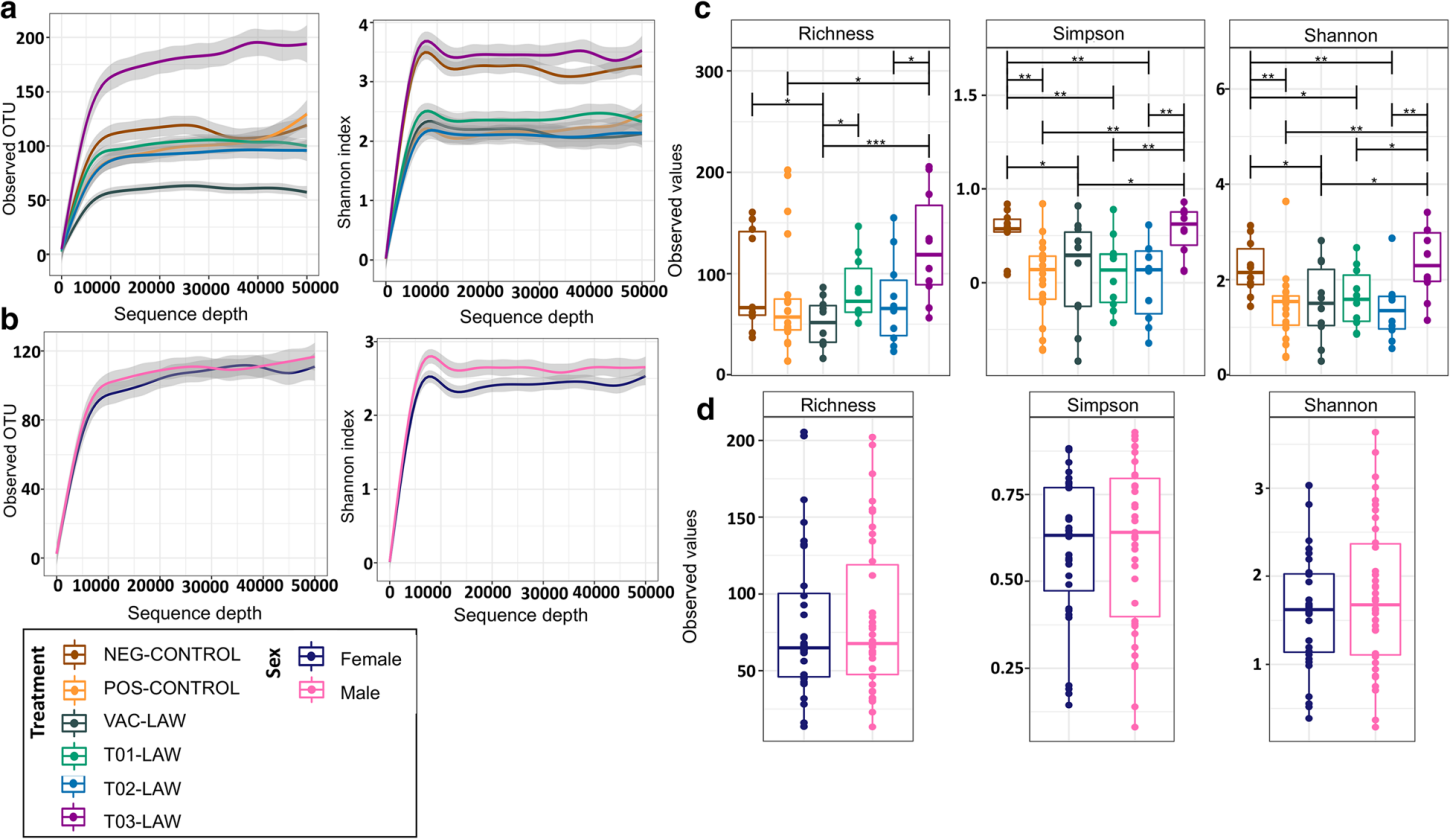
Changes in the ileal microbial structure associated with treatment groups. **a** Rarefaction curves of alpha diversity indices, i.e. observed OTUs and Shannon index, grouped by treatment. **b** Rarefaction curves of alpha diversity indices grouped by gender. **c** Boxplots of pairwise comparison of the treatment alpha diversity indices using Wilcoxon’s non-parametric test where asterisks represent an adjusted *P* value less than 0.05*, 0.001**, 0.0001***. **d** Boxplots of the gender alpha diversity indices

From the tissue homogenate used for inoculation of the pigs, a total of 127 bacterial taxa were identified of which 108/127 taxa contributed less than 1% of the overall population (Additional file 3). *L. intracellularis* accounted for 37.5% of the taxonomic abundances in the  inoculum. From the 70 ileal content samples, 4.4 million sequences were generated, 89% of which had a quality score of Q30 and above. Preliminary assessment without a sequence depth filter showed that the sequences clustered into 2241 unique OTUs with an average frequency of 57,237. At an even sampling depth of 10,000, 69 samples and 1025 unique OTUs were retained, and their distribution according to treatment is shown in Fig. 1. The refraction curves in Fig. 1a indicate that T03-LAW pigs and the NEG-CONTROL pigs had a higher diversity (Shannon index); indeed, the former had nearly four times the observed OTU count as compared to the VAC-LAW group. Similarly, and as indicated in Fig. 1b, ileal content from male pigs appeared to have a higher diversity (Shannon index); however, this was not statistically significant compared to female pigs.

### Microbiota structure of the ileum

#### Alpha diversity

The mean species richness, Simpson index, and Shannon index across all groups were 93, 0.53 and 1.9, respectively. A comparison between groups showed significant differences (*p* < 0.001) in all three alpha diversity indices as shown in Fig. 1c except richness between NEG-CONTROL and POS-CONTROL groups. A significant difference in diversity was evident between NEG-CONTROL and POS-CONTROL pigs. However, the largest differences were observed between T03-LAW and POS-CONTROL. Although significant differences in richness existed between T03-LAW and VAC-LAW for the  Shannon index, the same was not true for the Simpson index. There were no statistical differences between male and female pigs (Fig. 1d). Overall these results suggest that treatment was associated with an individual’s microbiota variations, however there was no impact of sex.

#### Beta diversity

**Fig. 2:**
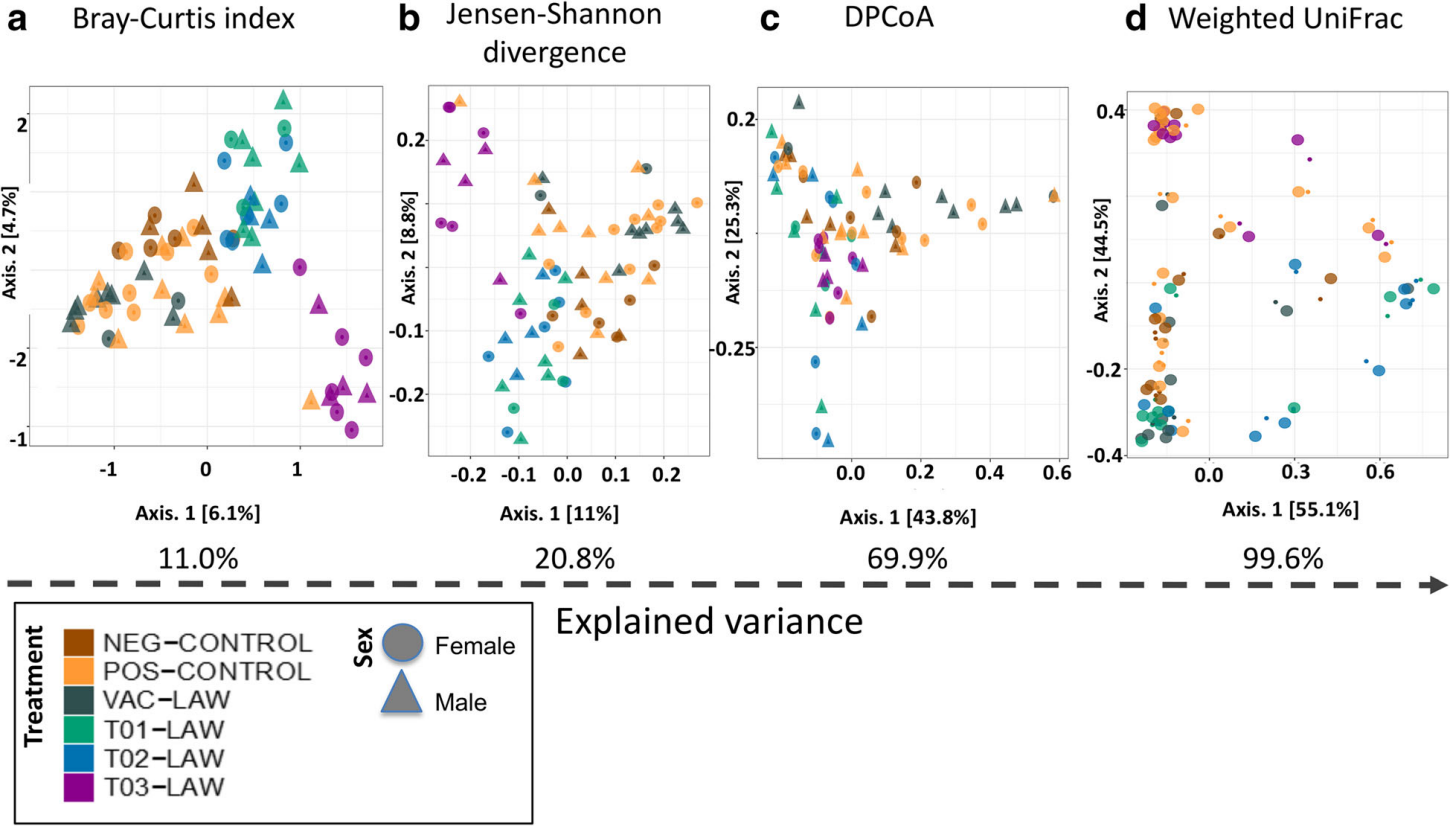
Comparative analysis between beta diversity indices and treatment groups. **a-c** Indication of the level of clustering and how treatment groups map to gender. **d** The level of clustering by treatment as a proxy for how much variation is explained by treatment group

Distinct clustering, corresponding to treatment, disappeared when increasing the total explained variance (Fig. 2). For example, clustering disappears at 69.9% (Fig. 2c), but is discernable between 11 and 20.8% (Fig. 2a and b), suggesting that the upper limit of variance attributable to treatment groups is between 20.8 and 69.9%. Note here that clustering is much more defined for pigs in the T03-LAW group, while T01-LAW and T02-LAW share a cluster, and the NEG-CONTROL, POS-CONTROL and VAC-LAW groups share the remaining cluster. The multivariable analysis of association was therefore restricted to the first three beta diversity indices.

The PerMANOVA analysis (Table 2) indeed shows that treatment as an experimental variable explained 26.8, 28.8 and 35.7% of the variation in microbiota structure as estimated using the Bray-Curtis index, Jensen-Shannon divergence, and DPCoA distances, respectively. This agrees with the univariate analysis in Fig. 2 and suggests that treatment explained 27–36% of the microbial structural variation. Therefore, since treatment here is associated with changes in alpha and/or and beta diversities, treatment significantly impacted structural changes in the ileal microbiota.

**Table 2:** Results of the PerMANOVA analysis using beta diversity indexes

	Bray-Curtis			Jensen-Shannon divergence			DPCoA distances		
	DF	*R*^2^	*P*-value	DF	*R*^2^	*P*-value	DF	*R*^2^	*P*-value
**Experimental factor**									
Treatment	5	0.268	0.0001	5	0.357	0.0001	5	0.288	0.0001
Sex	2	0.006	0.8063	2	0.006	0.6516	2	0.008	0.513
**Model**									
Residuals	63	0.72		63	0.635		63	0.703	
Total	69	1.00		69	1.00		69	1.00	

### Taxonomic profiles of ileum microbiota

**Fig. 3:**
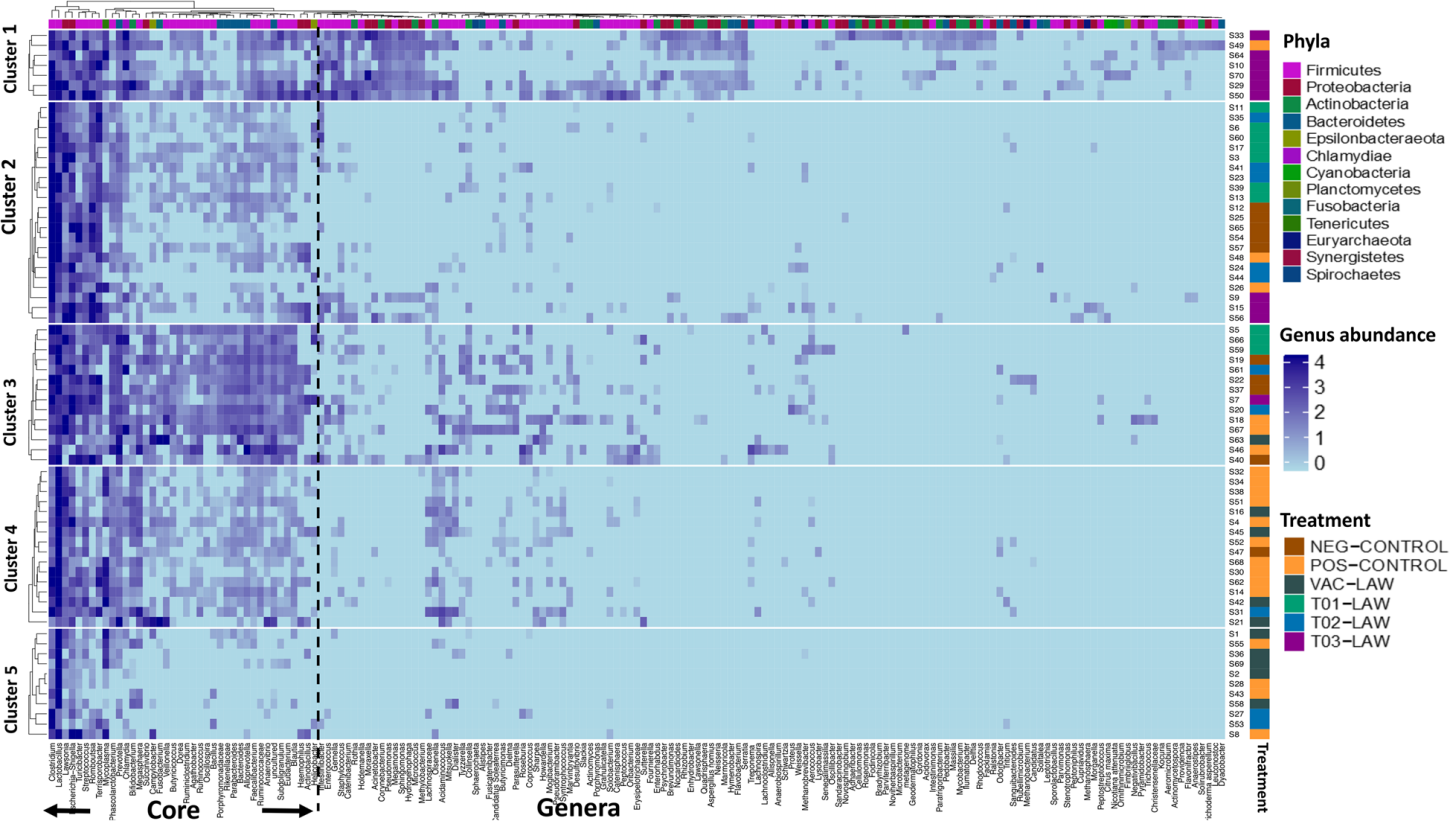
The genus level microbial richness characterized in this study clustered by abundance levels. The clusters are linked to the treatment groups and sex. Note that the first 38 genera on the x-axis represent the core i.e. genera present in at least 85% of the individuals

The 1025 OTUs clustered into 14 phyla, 85 families and 178 genera. At the phylum level *Firmicutes* and *Bacteriodetes* accounted for more than 75% of the abundance, other phyla included *Proteobacteria* and *Actinobacteria*. At the family level, *Ruminococcaceae*, *Prevotellaceae*, *Lachnospiraceae*, *Clostridiaceae*, and *Lactobacillaceae* were the most abundant. The heat trees in Additional file 4 show the phylogenetic relationships of ileal microbiota by treatment i.e. thick dark green branches represent the core microbes. T03-LAW had an overall enrichment shown as thick dark green branches through the heat tree (Additional file 4), indeed T03-LAW accounted for 87% of the OTUs and genera observed in this study. On more granular or closer analysis, the enrichment exhibited by T03-LAW is discernible as cluster 1 (Fig. 3), although a few T03-LAW pigs are also present in clusters 2 and 3 (Fig. 3). The core, i.e. genera observed in 85% of the samples, was composed of 38 genera including *Clostridium, Lawsonia, Lactobacillus, Streptococcus, Bifidobacterium* and others. In this regard we also observed that clusters 1 and 3 had an enriched core whereas cluster 5 had a depleted core (Fig. 3). In summary, clustering by taxonomic abundance was mainly associated with T03-LAW, which also exhibited the highest taxonomic diversity.

### Ileal microbiota and clinical characteristics of *L. intracellularis*

**Fig. 4:**
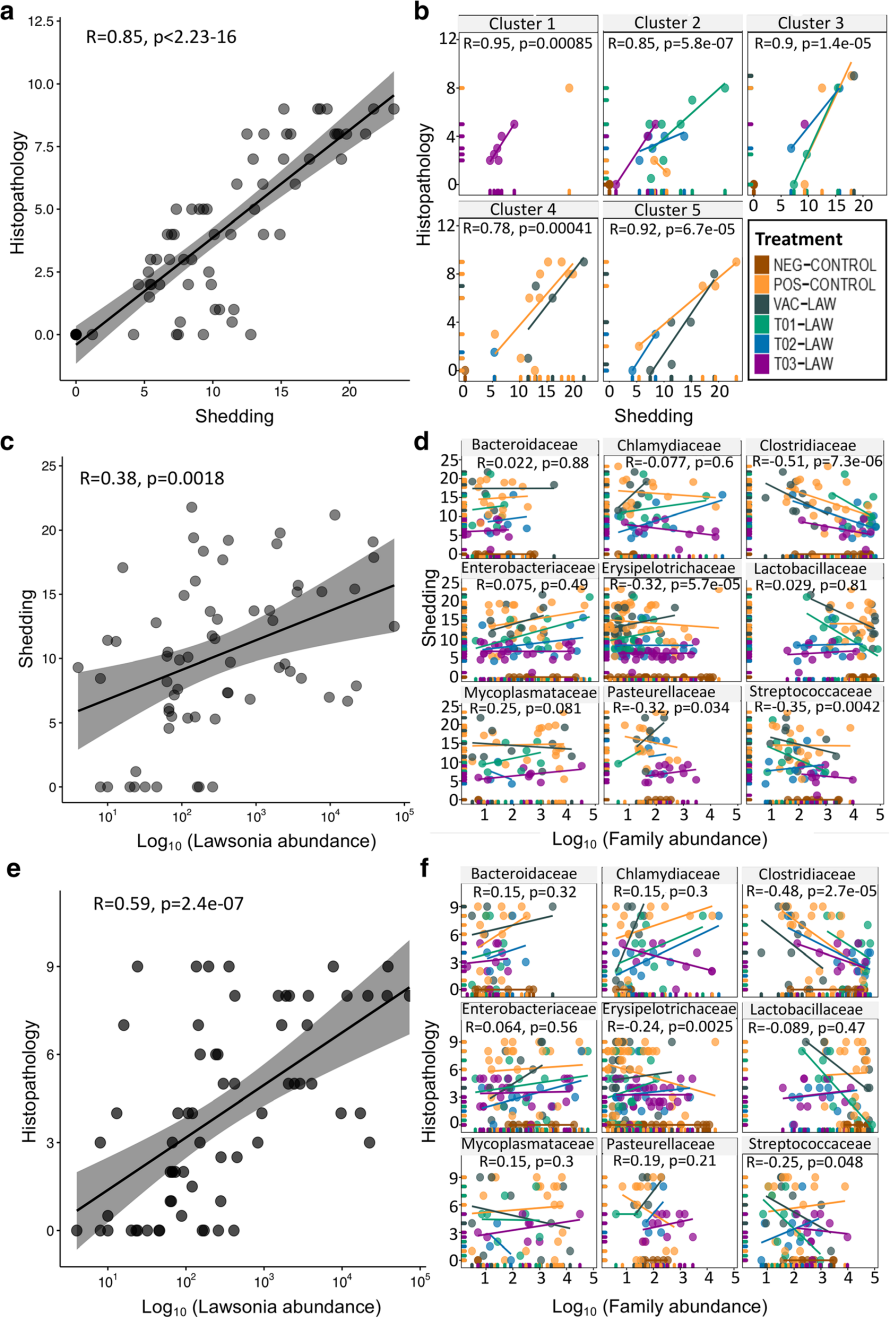
Association between *Lawsonia intracellularis* phenotypes i.e. histopathology and shedding and ilea microbiota taxonomic abundance (log_10_) at the family level. All treatment groups are color coded. R represents the correlation coefficient and the corresponding *P* value. **a** shows the relationship between shedding and pathology (microscopic ileum score) and **b** shows how this varies depending on taxonomic clustering and treatment group. **c** Overall comparison of shedding (log_10_) and microbial abundance. **d** Same relationship as in **A** faceted by selected number of microbial families (full profile in Additional files 4 and 5). **e** Overall comparison of pathology (microscopic ileum score) and microbial abundance. **f** Same relationship as in **c** faceted by selected families

**Fig. 5:**
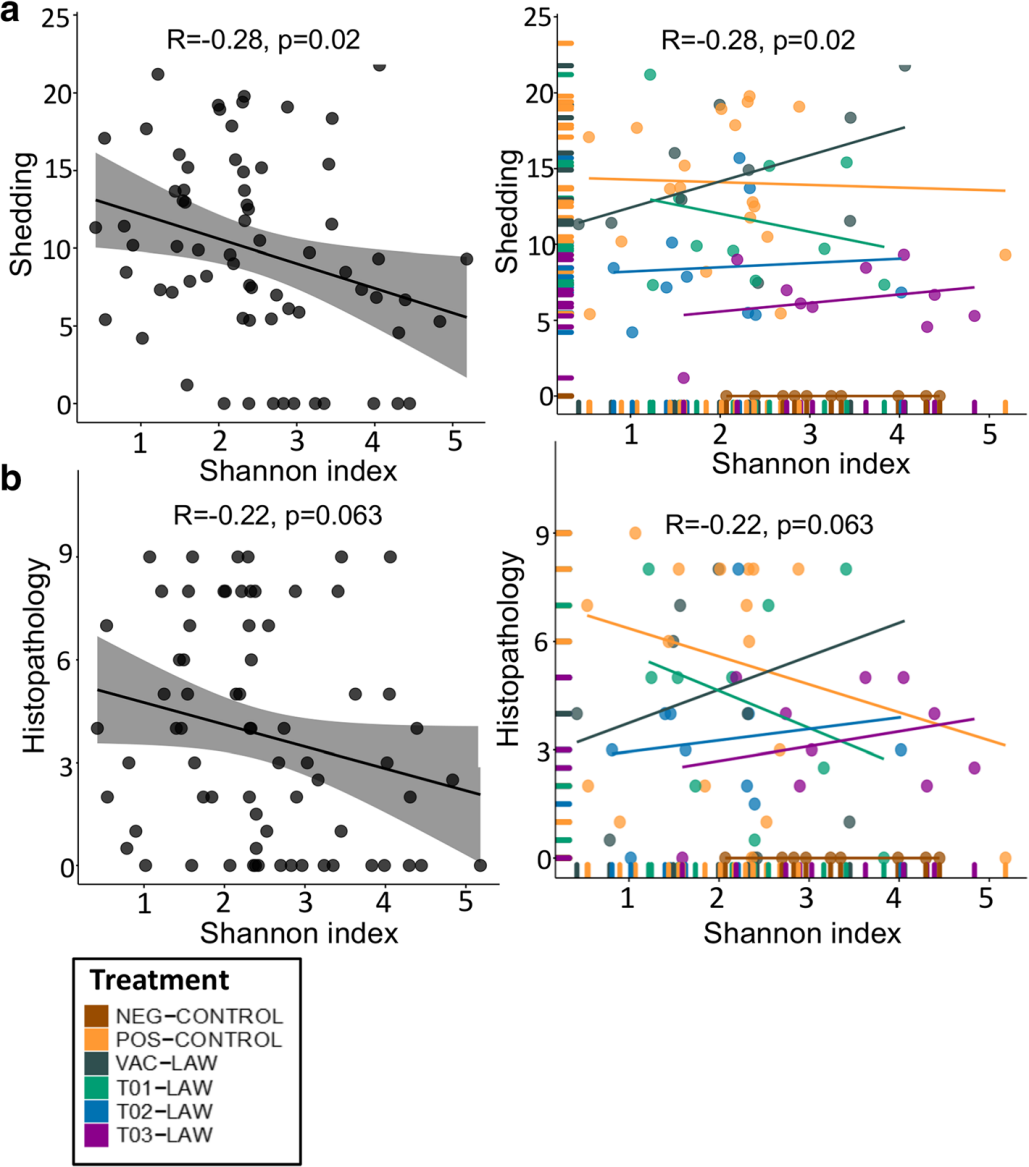
Relationship between *L. intracellularis* phenotype, including **a** shedding and **b** histopathology (microscopic ileum score), and their corresponding ileal microbiota diversity. Groups are colored by treatment and R represents the correlation coefficient and the corresponding *p* value

A direct correlation between histopathology in the ileum and fecal shedding of *L. intracellularis* is demonstrated in Fig. 4a. This relationship varied for the individuals in each of the five clusters defined in Fig. 3. For example, the highest diversity is present in cluster 1 (almost exclusively made up by T03-LAW), and core enrichment is present in cluster 3 whereas core depletion can be noted in cluster 5. In general, cluster 1 was associated with lower histopathology scores and shedding. It appears that changing the core genera population does not directly lead to a change in clinical presentation. Cluster 4 was dominated by members of the POS-CONTROL group. Generally, there was a discernible relationship between microbial abundance and clinical characteristics (shedding and histopathology) (Fig. 4c and e), however at the family level, there were significant correlations with *Clostridiaceae*, *Erysipelotrichacea* and *Streptococeae* (Fig. 4d and f). The direction of these correlations varied, for example an inverse correlation was seen between clinical characteristics and the abundance of *Clostridiaceae* for all treatment groups except for the NEG-CONTROL group which showed a flat (neutral) correlation for shedding and the histopathology score. The unit increase in abundance of *Streptoccocaeae* and *Erysipelotrichacea* was associated with a 0.25–0.35 and 0.24–0.32 log reduction in shedding and histopathology respectively (Fig. 4d and f). In groups T01-LAW, T03-LAW and VAC-LAW the abundance of *Streptococcaceae* was inversely correlated to shedding and histopathology, whereas the NEG-CONTROL group as well as the POS-CONTROL group, when looking at shedding only, showed no correlation with clinical signs. The opposite seems to be true for *Erysipelotrichacea,* where only T02-LAW and T03-LAW show a neutral correlation whereas the POS-CONTROL is inversely correlated. An inverse correlation was also observed for *Rummincococeae* (T01-LAW, T02-LAW, T03-LAW, VAC-LAW), *Coriobacteriaceae* (T01-LAW, T02-LAW, T03-LAW, VAC-LAW, POS-CONTROL for shedding and family abundance only) and *Peptostreptococaceae* (T01-LAW, T02-LAW, T03-LAW for histology and family abundance only, VAC-LAW, POS-CONTROL) (Additional files 5 and 6). An inverse correlation between shedding and the diversity of the ileal microbiota was identified (Fig. 5a). Indeed, a unit increase in the Shannon index was associated with a 2.8 log reduction of *L. intracellular is* DNA copy numbers shed in the feces. The direction of this relation was heavily influenced by T01-LAW. Although T03-LAW, T02-LAW and VAC-LAW exhibit a direct relationship, T03-LAW and to some extent also T02-LAW were characterized by lower levels of shedding. Critically, for shedding both control groups exhibit a neutral relationship between clinical characteristics and diversity which suggests that the observed effect is due to the treatment. It is noteworthy that the correlation between histopathology and diversity was not statistically significant (*p* = 0.063) but the trends were similar compared to shedding.

## Discussion

In this study, ileum microbiota characteristics in *L. intracellularis* infected pigs under different experimental treatment regiments were defined and characterized. We specifically compared possible prophylactic interventions to prevent *L. intracellularis* associated disease as opposed to traditional therapeutic methods such as antibiotic treatment. In future it may be worthwhile to repeat this study and to add a traditional antimicrobial treatment as an additional group. The ileum, as the primary site of *L. intracellularis* infection in pigs [38], was the selected anatomical location in this study. Our approach consisted of collecting ileum content from 9-week-old pigs, 16 days after experimental infection with *L. intracellularis* and 44 days post commercial *L. intracellularis* vaccination (VAC-LAW) or the start of three different probiotics (T01-LAW, T02-LAW, T03-LAW) which were added to the regular base diet given to pigs in all groups. This differs from other *L. intracellularis* studies focusing on the enteric microbiome which investigated fecal [11] or caecum and colon microbiota [16], or analyzed the distal ileum content using terminal restriction fragment length polymorphism [15] and provides a broad snapshot of *L. intracellularis* infection dynamics at the site of colonization. We specifically examined the bacterial community as earlier *L. intracellularis* experiments in gnotobiotic pigs suggested that a certain microbial background is required for *L. intracellularis* infection to establish [38]. The factor “treatment” was used to analyze the changes in ileum microbiota using 16S rRNA gene sequencing. We then linked observed changes to clinical phenotypes of *L. intracellularis* infection including fecal shedding and histopathology in the ileum. Overall, we observed a strong association between treatment and changes in ileum microbiota structure based on alpha diversity indices. These changes were significant and not limited to richness (i.e. the number of unique OTUs) but also to evenness and relative abundance. For example, richness was highest in T03-LAW and lowest in VAC-LAW (Fig. 1c). However, VAC-LAW had a higher average Simpson index compared to T01-LAW even though the unique OTU count in VAC-LAW was significantly lower. It remains puzzling why, in this study, the vaccinated group had the lowest OTU count but at the same time had a more even distribution of bacteria and how this impacted the overall outcome. A more even and diverse ileum microbiota may be beneficial for pigs infected with *L. intracellularis* as has been observed in relation with other pathogens [39, 40]. Microbiota changes were associated with specific clinical phenotypes as shown in the downstream analysis; specifically, increased diversity was associated with reduced histopathology lesions and fecal shedding. It is likely that the presence of specific organisms in T03-LAW pigs resulted in the desired effect on clinical presentation. It has been previously shown that AUC measurements and shedding duration were significantly increased in VAC-LAW pigs compared to T02-LAW and T03-LAW pigs; 40% of the VAC-LAW pigs had severe microscopic ileum lesions compared to 10% of T02-LAW and 0% of T03-LAW animals [24]. The reason for these previous observations is unknown. T01 was supplemented with *B. amyloliquefaciens*, T02 was supplemented with *B. licheniformis*, and T03 was given *B. pumilus*. While these all belong to the *Bacillus* species, it is well acknowledged in the probiotic literature that even members of the same species, i.e., different strains, differ in their properties and utility as a probiotics [41]. Additional in vivo but also in vitro studies are required to confirm the obtained results and to define the effect/mechanism of each of these *Bacillus* species on the microbiome and more specifically on *L. intracellularis* to identify possible mechanisms leading to reduction of *L. intracellularis* infection, shedding and lesions.

Similarly, we exploited the subtle methodological differences in analyzing beta diversity to define a range of variation likely attributable to treatment. Specifically, in this study, treatment explained between 26 and 36% of the population variation. To put this in context, the effect size of probiotic treatment is similar to the effect size of bile acid on the intestinal microbiome [42]. In this regard, the largest change in beta diversity was seen for T03-LAW while changes for T01-LAW and T02-LAW were similar, hence the co-clustering.

Our approach assumed that clustering by richness and abundance corresponding to treatment is indicative of a strong taxonomic effect for the experiment. The extent to which treatment impacted the ileal microbial structure differed for each group. These differences potentially define the exploitable usefulness of these treatments, specifically in augmenting clinical outcomes to control *L. intracelluaris* in pig herds. For example, significant differences were observed between POS-CONTROL and NEG-CONTROL for the alpha diversity indices and  discernible clusters were identified for the beta diversity indices. However, intriguingly, the microbial structural changes between POS-CONTROL and VAC-LAW were comparable. Members of the T03-LAW almost exclusively grouped in cluster 1 (Fig. 3) which accounts for a considerable amount of the overall observed richness. This dramatic increase in richness was associated with lower shedding and lower histopathology scores. T03-LAW appears to break the direct relationship (positive correlation) observed in all other *L. intracellularis* infected pigs between shedding and histopathology. The mechanisms for this are currently unknown but among other reasons could be due to a direct interaction of probiotic bacteria (or their metabolites) with either *L. intracellularis,* the established microbiome, or both. Alternatively, other microorganisms, induced by the probiotic bacteria or their metabolites, could have a major impact. Also, the probiotic bacteria could directly affect the protective biofilm formation on the enteric mucosal surface, such as changing the redox potential, resulting in an unfavorable environment for *L. intracellularis*. It may also be beneficial to increase the microbiota richness for the robustness of a niche in warding off a pathogen invasion. It is recognized that enteric pathogens, including *L. intracellularis,* have the potential to alter the bile acid metabolism [43]. Physiologically, 95% of bile salts are reabsorbed by the jejunum and ileum epithelium by active and passive transport and the small intestinal microbiome plays a major role in the bile acid metabolism [44]. Hence, it is likely that the bile acid metabolism is affected by the damage to the small intestinal epithelium due to *L. intracellularis* infection, the disruption of the physiological microbiome (dysbiosis) at that site brought on by the infection or both [15]. The favorable microbial composition and increased microbial diversity associated with probiotic treatment could mitigate *L. intracellularis* infection by impacting the bile acid metabolism and other similar physiological processes. In this particular context further research is required to identify the microbial combination of probiotics and mechanistic basis for these effects.

Shifts in the ileal core genera were associated with changes in the threshold for shedding level, i.e. the clusters with the most depleted core (cluster 5) and the most enriched cores (clusters 1 and 3). This suggests that depleting the ileal core genera results in a higher threshold for shedding. It is difficult to compare the core genera across studies. Besides age, commonly studies are done in pigs around weaning [45, 46], housing and diet may also impact core genera. Moreover, it has been recently determined that when defining the core microbiome in pigs, differences in study protocols have a significant impact and standardization of experimental techniques appear to be important [47]. Nevertheless, using a meta-analysis of 20 data sets, several shared genera such as *Prevotella*, *Clostridium*, *Alloprevotella*, and *Ruminococcus* were identified [47]. A more recent study, based on analysis of freshly collected feces, identified a pig core microbiome of 69 bacterial features present in all growth stages [48]. Besides confirming the earlier core genera findings, at the family level the top three families were *Prevotellaceae*, *Ruminococcaceae*, and *Lactobacillaceae* [48]. The analysis in this study does not suggest a major effect of the core microbiota on clinical presentation other than threshold differences in shedding detection (cluster 3 enriched and cluster 5 depleted in Fig. 4b).

In this study it was established that a direct relationship exists between pathology and shedding and identifying microbial factors that disrupt it provides a foundation for limiting the impact of this infection. The precise mechanism of this microbial interaction is unknown. The data obtained here will be useful to design future experiments in an attempt to further reduce bacterial shedding and pathology. This perhaps could advance probiotic supplementation to limit the impact of infectious diseases in food production. Limiting pathology arguably reduces not only the adverse effects on an animal’s productivity but also shedding and thus limits transmission to other pigs. In this regard, T03-LAW (cluster 1) appears to be the only treatment group in which the shedding dynamics were reduced. At the family level, a log increase in abundance of *Clostridiaceae*, *Streptoccaceae* or *Erysipelatricheae* was associated with 3.9, 3.1 and 3.4 log reduction in copies of *L. intracellularis* shedding and pathology rank, respectively. In particular, reduction in shedding and pathology in the VAC-LAW group appeared to be influenced by the increase in abundance of *Clostridiaceae*. A similar but weaker relationship was noted for T01-LAW and T02-LAW. In a previous study, analysis of the microbiota of *L. intracellular is* vaccinated pigs showed that vaccination led to changes in the abundance of Clostridium species, including *Clostridium butyricum* [11] which is in agreement with the results of this study. However, an increase in abundance of the three aforementioned families was not associated with shedding or pathology (reduction or increase) for T03-LAW.

An increase of one unit in the Shannon diversity index resulted in a 2.8 log reduction in shedding in this study. In other words, by increasing diversity one could reduce shedding of *L. intracellularis*. In addition to T03-LAW, a reduction in shedding associated with increased diversity was also evident for T01-LAW and T02-LAW. An increase in diversity was however associated with increased shedding among VAC-LAW pigs and this still requires an explanation. Perhaps this could reflect the effect of a longer term dysbiosis as the VAC-LAW group was first exposed to attenuated *L. intracellularis* before being exposed to pathogenic *L. intracellularis* challenge. In addition, the VAC-LAW group did manifest associated changes in the ileal microbiota after *L. intracellularis* challenge (Additional file 4) which warrants further studies. Crucially, the comparison between clinical phenotypes and ileal microbiota diversity showed that both control groups (NEG-CONTROL, POS-CONTROL) were not affected by this relationship. The direction and strength of the relationships observed with a treatment, i.e. the associated microbial community, therefore reflects its true impact.

In this study the pigs were not challenged with a pure *L. intracellularis* inoculum obtained by in vitro culture but rather with ileum mucosa scrapings, from a commercial vendor, containing *L. intracellularis* obtained from pigs deliberately infected under experimental conditions to produce the inoculum stock. This was done because in vitro propagation of *L. intracellularis* is difficult and only successfully performed in few labs worldwide [17, 49], wild-type strains are often attenuated after passaging [50], and clinical ileitis can be difficult to reproduce with pure cultured inoculum [51] with some exemptions [52]. The important comparisons in this study were between the T01-LAW, T02-LAW, T03-LAW and POS-CONTROL groups which all received an identical inoculum. 16 s rRNA gene amplicon sequencing obtained from the inoculum indicate that 37.5% of the stock was *L. intracellularis* whereas other bacteria where only present in small percentages (Additional file 3).

A potential application of the study findings includes microbiota augmentation to modify pathology, shedding and, by extension, *L. intracellularis* transmission. This may have profound implications for the dependence on antibiotic use to control infectious diseases in livestock production systems. However, extensive research will be required to optimize probiotics (and likely also prebiotics) outside the realm of controlled experimentation. Ultimately such scalable innovation means livestock will be raised without adverse environmental effects while also minimizing the contribution to the evolution of antibiotic resistance. In this regard, our findings indicate the potential usefulness of microbial families such as *Clostridiaceae, Streptoccaceae, Erysipelatricheae*, *Rummincococeae*, *Coriobacteriaceae* and *Peptostreptococaceae* in augmenting clinical outcomes during *L. intracellularis* infections.

## Conclusions

In this study, significant effects on the ileal microbiota structure were observed during experimental infection of pigs with *L. intracellularis,* either with prophylactic treatment in the form of a commercial live *L. intracellularis* vaccine or in-feed probiotics. These changes exhibited as taxonomic shifts and included, but were not limited to, core and accessory genera depletion or enrichment. We observed that microbial changes such as diversity and richness had an inverse correlation with clinical characteristics such as ileal histopathology and fecal shedding. The extent to which this relation manifested varied depending on treatment, which raises the potential for using microbial manipulation to decrease pathology and shedding. Modulating these clinical parameters appears to be the foundation to limiting disease transmission as well as promoting individual animal resilience to disease. Further studies need to be done to explore the causal relationship and underlying mechanism(s) of probiotic feed supplementation on *L. intracellularis* induced ileal lesions. To unravel the complex associations between microbial diversity and *L. intracellularis* infection/pathogenesis, a longitudinal pig study with suitable design, such as sampling at different time points by staggered sacrifice of pigs within a group, introduction of the probiotic feed intervention at different time points before or after *L. intracellularis* infection, or ileal content transfer in addition to other tools would also be required.

## Supplementary Information

**Additional file 1 **Number of positive pigs/total pigs per group (group mean ± SEM) for log10 L. intracellularis DNA in rectal swabs at different days post *L. intracellularis* challenge [24]. Different superscripts (^A,B,C^) indicate significantly different group means on a certain day.

**Additional file 2.** Association of histopathology and shedding for each of the treatment groups.

**Additional file 3.** Taxa and percentage of bacteria present the inoculum used to challenge the pigs.

**Additional file 4.** Taxonomic characteristics of ileal microbiota. OTUs clustered according to their phylogenetic relationship and colored by abundance, also called a heat-tree. The core microbes can be identified by the dark green backbone while the transient/accessory microbes compose the rest of the tree branches and change by treatment groups.

**Additional file 5.** Taxonomic association with shedding. The relationship between shedding and the abundance of individual families colored by treatment group is demonstrated.

**Additional file 6.** Taxonomic association with pathology (microscopic ileum score). The relationship between pathology and the abundance of individual families colored by treatment group is demonstrated.

## Data Availability

The datasets generated and/or analyzed during this study are available in the BioProject repository, http://www.ncbi.nlm.nih.gov/bioproject/660793.

## Abbreviations

16S rRNA: 16S ribosomal RNA
AUC: Area under the curve
bp: Base pair
BSL-2: Biosafety level 2
CFU: Colony forming unit
DNA: Deoxyribonucleic acid
dpc: Day post challenge
DPCoA: Double principal coordinate analysis
DF: Degree of freedom
g: Gram
JSD: Jensen-Shannon divergence
*L. intracellularis*: *Lawsonia intracellularis*
min: Minute
mL: Milliliter
NEG-CONTROL: Negative control group not treated or challenged
OTU: Operational taxonomic unit
PCR: Polymerase chain reaction
PerMANOVA: Permutation multivariate analysis of variance
POS-CONTROL: Positive control group, not treated but infected with *L. intracellularis*
sec: Second
T01-LAW: Supplemented with *B. amyloliquefaciens* and infected with *L. intracellularis*
T02-LAW: Supplemented with *B. licheniformis* and infected with *L. intracellularis*
T03-LAW: Supplemented with *B. pumilus* and infected with *L. intracellularis*
VAC-LAW: Vaccinated against *L. intracellularis* and infected with* L. intracellularis*
