# Supplementary figures and images for: Probiotics mediated gut microbiota diversity shifts are associated with reduction in histopathology and shedding of Lawsonia intracellularis
Source: Anim Microbiome. 2021 Mar 4;3:22. doi: 10.1186/s42523-021-00084-6 (PMC7931366; doi:10.1186/s42523-021-00084-6)

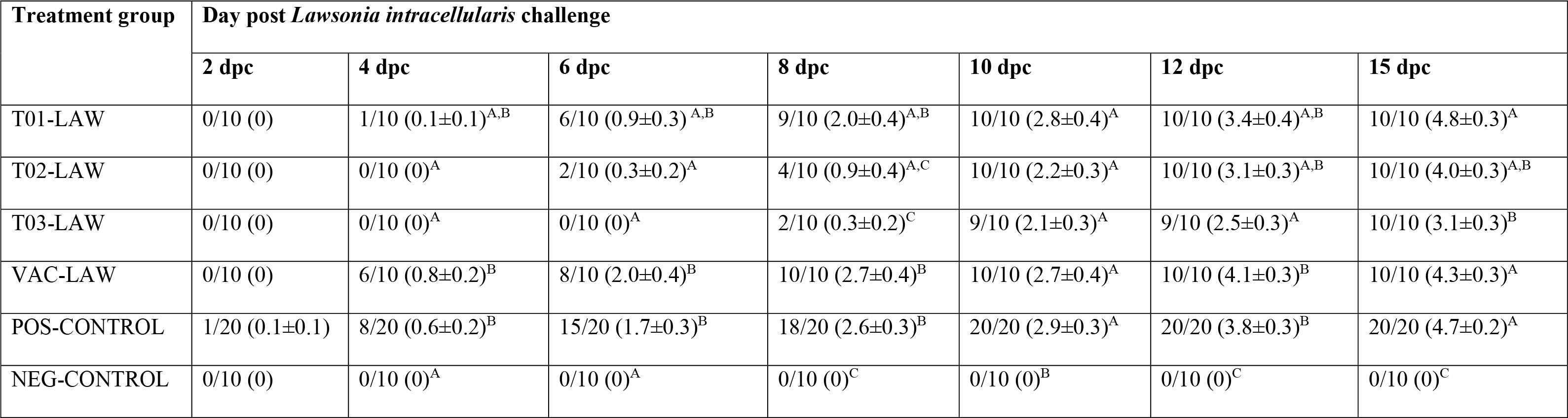

Supplement: Supplementary file 1 — Additional file 1 Number of positive pigs/total pigs per group (group mean ± SEM) for log10 L. intracellularis DNA in rectal swabs at different days post L. intracellularis challenge [24]. Different superscripts (A,B,C) indicate significantly different group means on a certain day. [file 42523_2021_84_MOESM1_ESM.tif]

Histopathology

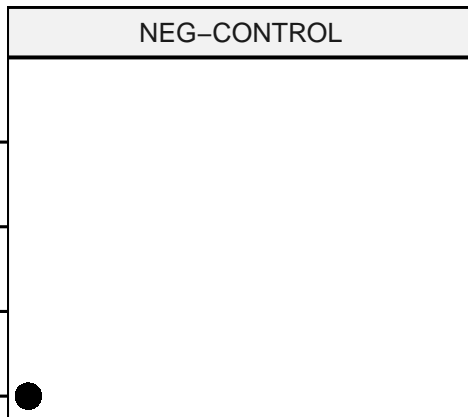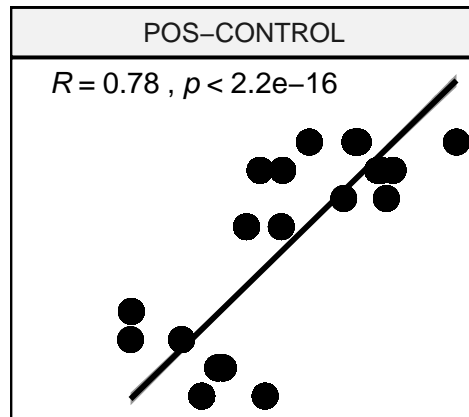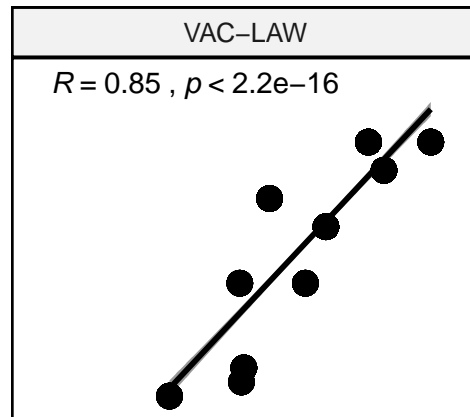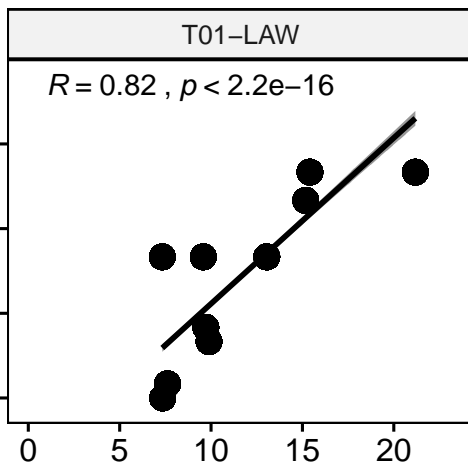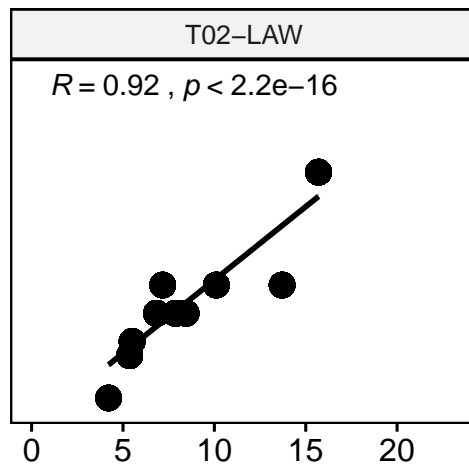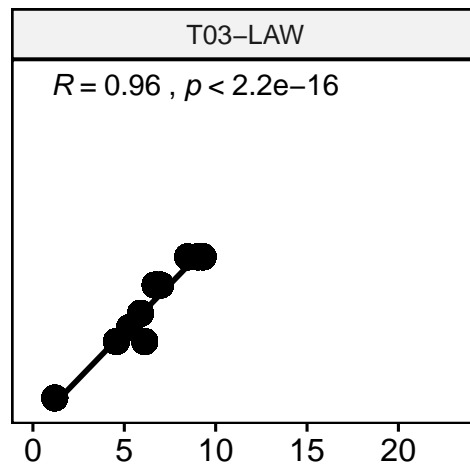

log10(Shedding)

Supplement: Supplementary file 2 — Additional file 2. Association of histopathology and shedding for each of the treatment groups. [file 42523_2021_84_MOESM2_ESM.pdf]

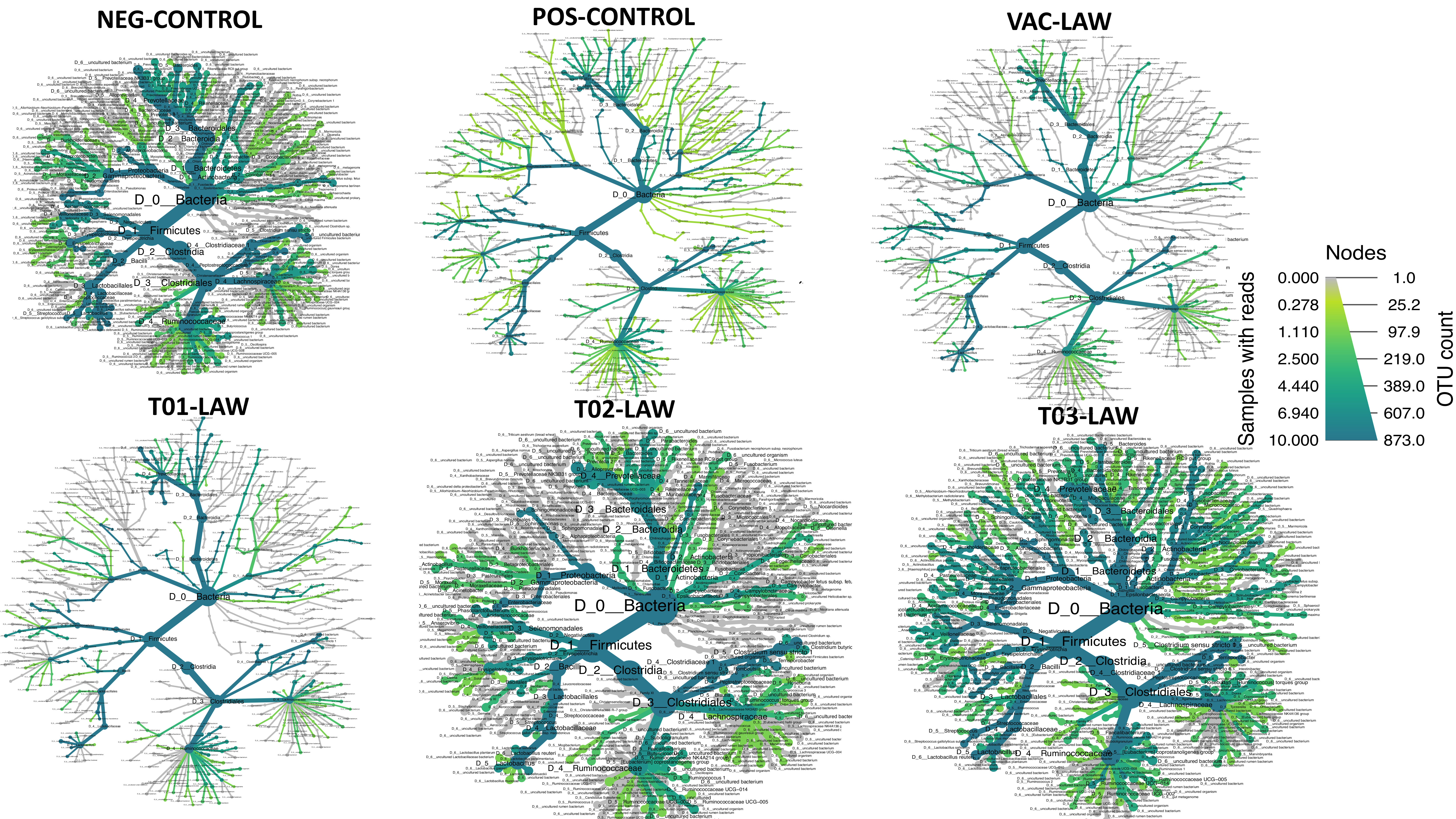

Supplement: Supplementary file 4 — Additional file 4. Taxonomic characteristics of ileal microbiota. OTUs clustered according to their phylogenetic relationship and colored by abundance, also called a heat-tree. The core microbes can be identified by the dark green backbone while the transient/accessory microbes compose the rest of the tree branches and change by treatment groups. [file 42523_2021_84_MOESM4_ESM.tif]

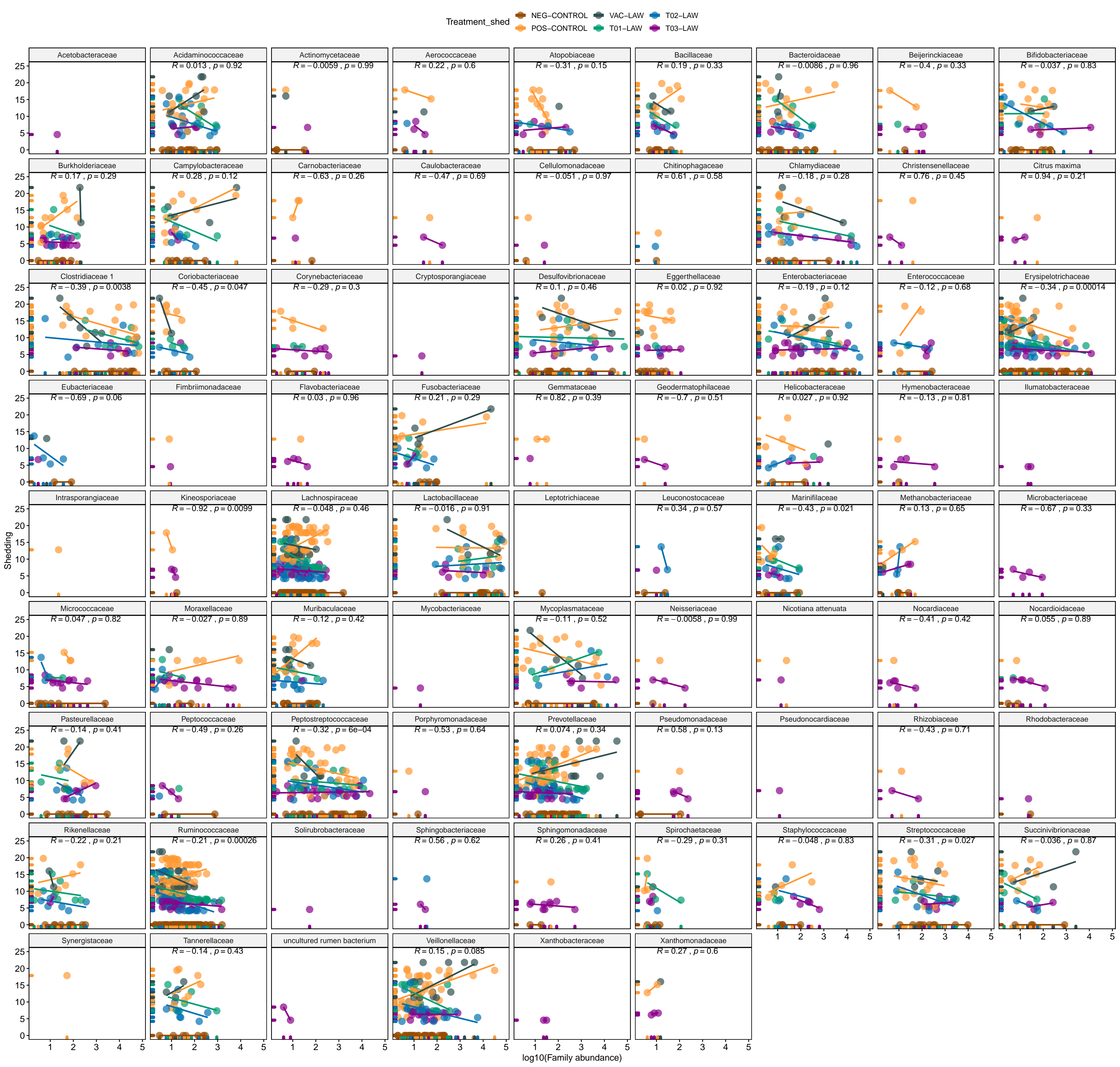

Supplement: Supplementary file 5 — Additional file 5. Taxonomic association with shedding. The relationship between shedding and the abundance of individual families colored by treatment group is demonstrated. [file 42523_2021_84_MOESM5_ESM.pdf]

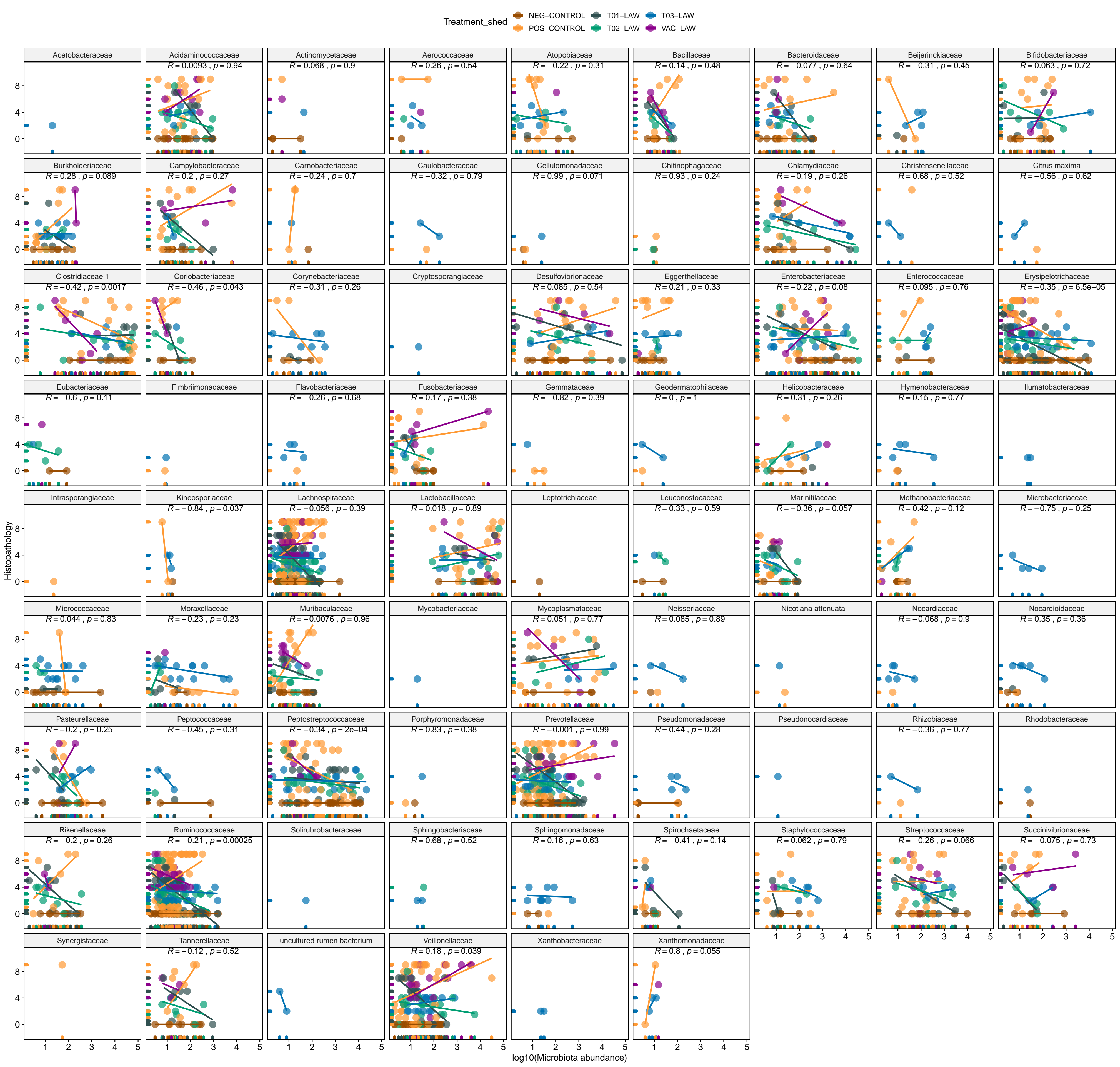

Supplement: Supplementary file 6 — Additional file 6. Taxonomic association with pathology (microscopic ileum score). The relationship between pathology and the abundance of individual families colored by treatment group is demonstrated. [file 42523_2021_84_MOESM6_ESM.pdf]
