# Supplementary material for: Probiotics mediated gut microbiota diversity shifts are associated with reduction in histopathology and shedding of Lawsonia intracellularis
Source: Anim Microbiome. 2021 Mar 4;3:22. doi: 10.1186/s42523-021-00084-6 (PMC7931366; doi:10.1186/s42523-021-00084-6)
Supplement: Supplementary file 3 — Additional file 3. Taxa and percentage of bacteria present the inoculum used to challenge the pigs. [file 42523_2021_84_MOESM3_ESM.pdf]

**Phylum**	**Order**	**Class**	**Family**	**Genus**	**Percentage**
Fibrobacteres	Fibrobacterales	Fibrobacteria	Fibrobacteraceae	Fibrobacter	0.001
Firmicutes	Clostridiales	Clostridia	Christensenellaceae	Christensenellaceae_R-7_group	0.001
Firmicutes	Clostridiales	Clostridia	Lachnospiraceae	Lachnospiraceae_UCG-004	0.001
Firmicutes	Clostridiales	Clostridia	Lachnospiraceae	uncultured	0.001
Firmicutes	Clostridiales	Clostridia	Peptostreptococcaceae	Terrisporobacter	0.001
Firmicutes	Clostridiales	Clostridia	Ruminococcaceae	Flavonifractor	0.001
Firmicutes	Erysipelotrichales	Erysipelotrichia	Erysipelotrichaceae	NA	0.001
Firmicutes	Clostridiales	Clostridia	Lachnospiraceae	Lachnospiraceae_ge	0.002
Firmicutes	Clostridiales	Clostridia	Peptostreptococcaceae	Intestinibacter	0.002
Actinobacteria	Coriobacteriales	Coriobacteriia	Coriobacteriaceae	NA	0.003
Firmicutes	Bacillales	Bacilli	NA	NA	0.003
Firmicutes	Clostridiales	Clostridia	Ruminococcaceae	Ruminococcaceae_UCG-003	0.003
Actinobacteria	Micrococcales	Actinobacteria	Microbacteriaceae	NA	0.003
Actinobacteria	Micrococcales	Actinobacteria	Micrococcaceae	Nesterenkonia	0.003
Firmicutes	Clostridiales	Clostridia	Ruminococcaceae	Ruminiclostridium_9	0.003
Firmicutes	Halanaerobiales	Clostridia	ODP1230B8.23	ODP1230B8.23_ge	0.003
Firmicutes	Erysipelotrichales	Erysipelotrichia	Erysipelotrichaceae	Turicibacter	0.003
Bacteroidetes	Bacteroidales	Bacteroidia	Prevotellaceae	Prevotellaceae_UCG-001	0.005
Bacteroidetes	Bacteroidales	Bacteroidia	Rikenellaceae	Alistipes	0.005
Lentisphaerae	Victivallales	Lentisphaeria	vadinBE97	vadinBE97_ge	0.005
Bacteroidetes	Bacteroidales	Bacteroidia	NA	NA	0.006
Firmicutes	Clostridiales	Clostridia	Ruminococcaceae	Anaerotruncus	0.006
Firmicutes	Lactobacillales	Bacilli	Enterococcaceae	Enterococcus	0.008
Firmicutes	Erysipelotrichales	Erysipelotrichia	Erysipelotrichaceae	uncultured	0.008
Proteobacteria	Xanthomonadales	Gammaproteobacteria	Xanthomonadaceae	NA	0.008
Bacteroidetes	Bacteroidales	Bacteroidia	Porphyromonadaceae	Parabacteroides	0.009
Proteobacteria	Aeromonadales	Gammaproteobacteria	Succinivibrionaceae	Succinivibrio	0.009
Firmicutes	Clostridiales	Clostridia	Family_XIII	Family_XIII_UCG-001	0.011

Firmicutes	Clostridiales	Clostridia	Lachnospiraceae	Coprococcus_3	0.011
Firmicutes	Clostridiales	Clostridia	Lachnospiraceae	Howardella	0.011
Firmicutes	Erysipelotrichales	Erysipelotrichia	Erysipelotrichaceae	Holdemanella	0.012
Proteobacteria	Pasteurellales	Gammaproteobacteria	Pasteurellaceae	Pasteurellaceae_ge	0.012
Spirochaetae	Spirochaetales	Spirochaetes	Spirochaetaceae	Sphaerochaeta	0.012
Actinobacteria	Micrococcales	Actinobacteria	Microbacteriaceae	Clavibacter	0.014
Actinobacteria	Corynebacteriales	Actinobacteria	Tsukamurellaceae	Tsukamurella	0.016
Actinobacteria	Micrococcales	Actinobacteria	Dermabacteraceae	Brachybacterium	0.016
Actinobacteria	Coriobacteriales	Coriobacteriia	Coriobacteriaceae	Enterorhabdus	0.017
Proteobacteria	Sphingomonadales	Alphaproteobacteria	Sphingomonadaceae	Sphingomonas	0.017
Firmicutes	Clostridiales	Clostridia	Lachnospiraceae	Tyzzerella	0.019
Firmicutes	Selenomonadales	Negativicutes	Veillonellaceae	Allisonella	0.019
Tenericutes	Mollicutes_RF9	Mollicutes	Mollicutes_RF9_fa	Mollicutes_RF9_ge	0.019
Firmicutes	Clostridiales	Clostridia	Lachnospiraceae	Acetitomaculum	0.021
Firmicutes	Clostridiales	Clostridia	Ruminococcaceae	Ruminococcus_2	0.021
Bacteroidetes	Bacteroidales	Bacteroidia	Bacteroidaceae	Bacteroides	0.027
Firmicutes	Clostridiales	Clostridia	NA	NA	0.027
Proteobacteria	Rhodobacterales	Alphaproteobacteria	Rhodobacteraceae	Paracoccus	0.027
Firmicutes	Clostridiales	Clostridia	Family_XIII	Family_XIII_ge	0.028
Firmicutes	Clostridiales	Clostridia	Lachnospiraceae	Lachnospiraceae_NK3A20_group	0.028
Proteobacteria	Burkholderiales	Betaproteobacteria	Alcaligenaceae	Sutterella	0.031
Verrucomicrobia	Verrucomicrobiales	Verrucomicrobiae	Verrucomicrobiaceae	Akkermansia	0.033
Deinococcus-Thermus	Thermales	Deinococci	Thermaceae	Thermus	0.035
Firmicutes	Bacillales	Bacilli	Staphylococcaceae	Staphylococcus	0.037
Firmicutes	Erysipelotrichales	Erysipelotrichia	Erysipelotrichaceae	Erysipelotrichaceae_UCG-002	0.039
Proteobacteria	Burkholderiales	Betaproteobacteria	Comamonadaceae	NA	0.041
Firmicutes	Clostridiales	Clostridia	Ruminococcaceae	Ruminococcaceae_UCG-004	0.042
Actinobacteria	Corynebacteriales	Actinobacteria	Corynebacteriaceae	Corynebacterium_1	0.044

Firmicutes	Erysipelotrichales	Erysipelotrichia	Erysipelotrichaceae	Catenibacterium	0.045
Bacteroidetes	Bacteroidales	Bacteroidia	Prevotellaceae	Prevotella_1	0.047
Proteobacteria	Pseudomonadales	Gammaproteobacteria	Moraxellaceae	Acinetobacter	0.047
Bacteroidetes	Bacteroidales	Bacteroidia	Porphyromonadaceae	Odoribacter	0.049
Firmicutes	Clostridiales	Clostridia	Family_XIII	Mogibacterium	0.049
Bacteroidetes	Bacteroidales	Bacteroidia	Rikenellaceae	Rikenellaceae_RC9_gut_group	0.051
Firmicutes	Clostridiales	Clostridia	Family_XIII	Family_XIII_AD3011_group	0.060
Firmicutes	Clostridiales	Clostridia	Ruminococcaceae	Ruminococcaceae_UCG-014	0.068
Actinobacteria	Coriobacteriales	Coriobacteriia	Coriobacteriaceae	Collinsella	0.079
Bacteroidetes	Cytophagales	Cytophagia	Cytophagaceae	Leadbetterella	0.080
Actinobacteria	Bifidobacteriales	Actinobacteria	Bifidobacteriaceae	Bifidobacterium	0.094
Firmicutes	Clostridiales	Clostridia	Lachnospiraceae	Dorea	0.094
Firmicutes	Clostridiales	Clostridia	Lachnospiraceae	Blautia	0.098
Bacteroidetes	Bacteroidales	Bacteroidia	Prevotellaceae	NA	0.104
Firmicutes	Clostridiales	Clostridia	Ruminococcaceae	NA	0.104
Firmicutes	Clostridiales	Clostridia	Peptostreptococcaceae	NA	0.108
Firmicutes	Clostridiales	Clostridia	Clostridiaceae_1	Clostridium_sensu_stricto_1	0.110
Firmicutes	Clostridiales	Clostridia	Ruminococcaceae	Ruminococcus_1	0.114
Bacteroidetes	Bacteroidales	Bacteroidia	Porphyromonadaceae	Butyricimonas	0.119
NA	NA	NA	NA	NA	0.122
Firmicutes	Clostridiales	Clostridia	Ruminococcaceae	Ruminococcaceae_UCG-008	0.122
Proteobacteria	Pseudomonadales	Gammaproteobacteria	Pseudomonadaceae	Pseudomonas	0.123
Bacteroidetes	Cytophagales	Cytophagia	Cytophagaceae	Hymenobacter	0.128
Firmicutes	Selenomonadales	Negativicutes	Veillonellaceae	uncultured	0.139
Firmicutes	Clostridiales	Clostridia	Ruminococcaceae	Faecalibacterium	0.149
Firmicutes	Erysipelotrichales	Erysipelotrichia	Erysipelotrichaceae	Sharpea	0.161
Proteobacteria	Aeromonadales	Gammaproteobacteria	Succinivibrionaceae	uncultured	0.173
Firmicutes	Clostridiales	Clostridia	Ruminococcaceae	Ruminococcaceae_UCG-002	0.176
Firmicutes	Clostridiales	Clostridia	Clostridiales_vadinBB60_group	Clostridiales_vadinBB60_group_ge	0.199

Firmicutes	Clostridiales	Clostridia	Lachnospiraceae	Roseburia	0.217
Bacteroidetes	Bacteroidales	Bacteroidia	Prevotellaceae	Prevotellaceae_UCG-003	0.223
Firmicutes	Clostridiales	Clostridia	Ruminococcaceae	Butyricicoccus	0.233
Firmicutes	Clostridiales	Clostridia	Ruminococcaceae	Ruminococcaceae_UCG-005	0.237
Firmicutes	Clostridiales	Clostridia	Ruminococcaceae	Ruminococcaceae_ge	0.288
Chlamydiae	Chlamydiales	Chlamydiae	Chlamydiaceae	Chlamydia	0.301
Bacteroidetes	Bacteroidales	Bacteroidia	Rikenellaceae	dgA-11_gut_group	0.327
Firmicutes	Clostridiales	Clostridia	Ruminococcaceae	Ruminococcaceae_NK4A214_group	0.337
Spirochaetae	Spirochaetales	Spirochaetes	Spirochaetaceae	Treponema_2	0.365
Proteobacteria	Desulfovibrionales	Deltaproteobacteria	Desulfovibrionaceae	Desulfovibrio	0.387
Proteobacteria	Enterobacteriales	Gammaproteobacteria	Enterobacteriaceae	NA	0.402
Firmicutes	Clostridiales	Clostridia	Ruminococcaceae	Subdoligranulum	0.425
Proteobacteria	Campylobacterales	Epsilonproteobacteria	Helicobacteraceae	Helicobacter	0.426
Firmicutes	Clostridiales	Clostridia	Lachnospiraceae	NA	0.438
Actinobacteria	Coriobacteriales	Coriobacteriia	Coriobacteriaceae	Olsenella	0.442
Bacteroidetes	Bacteroidales	Bacteroidia	Prevotellaceae	uncultured	0.515
Tenericutes	Mycoplasmatales	Mollicutes	Mycoplasmataceae	Mycoplasma	0.624
Bacteroidetes	Bacteroidales	Bacteroidia	Prevotellaceae	Prevotellaceae_NK3B31_group	0.630
Bacteroidetes	Bacteroidales	Bacteroidia	Bacteroidales_S24-7_group	Bacteroidales_S24-7_group_ge	0.645
Proteobacteria	Aeromonadales	Gammaproteobacteria	Succinivibrionaceae	NA	0.724
Bacteroidetes	Bacteroidales	Bacteroidia	Prevotellaceae	Prevotella_2	0.874
Bacteroidetes	Bacteroidales	Bacteroidia	Prevotellaceae	Alloprevotella	0.892
Firmicutes	Lactobacillales	Bacilli	Streptococcaceae	Streptococcus	0.945
Proteobacteria	Rhizobiales	Alphaproteobacteria	Methylobacteriaceae	Methylobacterium	1.015
Proteobacteria	Pasteurellales	Gammaproteobacteria	Pasteurellaceae	Actinobacillus	1.054
Firmicutes	Selenomonadales	Negativicutes	Veillonellaceae	Mitsuokella	1.076
Firmicutes	Selenomonadales	Negativicutes	Veillonellaceae	Veillonella	1.304
Fusobacteria	Fusobacteriales	Fusobacteriia	Fusobacteriaceae	Fusobacterium	1.415
Firmicutes	Selenomonadales	Negativicutes	Veillonellaceae	Anaerovibrio	1.717

Firmicutes	Selenomonadales	Negativicutes	Acidaminococcaceae	Phascolarctobacterium	1.727
Proteobacteria	Campylobacterales	Epsilonproteobacteria	Campylobacteraceae	Campylobacter	1.888
Proteobacteria	Enterobacteriales	Gammaproteobacteria	Enterobacteriaceae	Escherichia-Shigella	1.958
Firmicutes	Selenomonadales	Negativicutes	Acidaminococcaceae	Acidaminococcus	2.158
Firmicutes	Lactobacillales	Bacilli	Lactobacillaceae	Lactobacillus	2.164
Bacteroidetes	Bacteroidales	Bacteroidia	Prevotellaceae	Prevotella_7	2.185
Firmicutes	Selenomonadales	Negativicutes	Veillonellaceae	Dialister	2.731
Firmicutes	Selenomonadales	Negativicutes	Veillonellaceae	NA	4.346
Firmicutes	Selenomonadales	Negativicutes	Veillonellaceae	Selenomonas	5.548
Bacteroidetes	Bacteroidales	Bacteroidia	Prevotellaceae	Prevotella_9	6.752
Firmicutes	Selenomonadales	Negativicutes	Veillonellaceae	Megasphaera	8.793
Proteobacteria	Desulfovibrionales	Deltaproteobacteria	Desulfovibrionaceae	Lawsonia	37.478
